# Progression events defined by home-based assessment of motor function in multiple sclerosis: protocol of a prospective study

**DOI:** 10.3389/fneur.2023.1258635

**Published:** 2023-10-10

**Authors:** Eva-Maria Dorsch, Hanna Marie Röhling, Dario Zocholl, Lorena Hafermann, Friedemann Paul, Tanja Schmitz-Hübsch

**Affiliations:** ^1^Experimental and Clinical Research Center, a Cooperation between the Max-Delbrück-Center for Molecular Medicine in the Helmholtz Association and the Charité—Universitätsmedizin Berlin, Berlin, Germany; ^2^Experimental and Clinical Research Center, Charité—Universitätsmedizin Berlin, Corporate Member of Freie Universität Berlin and Humboldt-Universität zu Berlin, Berlin, Germany; ^3^Max-Delbrück-Center for Molecular Medicine in the Helmholtz Association (MDC), Berlin, Germany; ^4^Department of Neurology, Charité—Universitätsmedizin Berlin, Corporate Member of Freie Universität Berlin and Humboldt Universität zu Berlin, Berlin, Germany; ^5^Neuroscience Clinical Research Center, Charité—Universitätsmedizin Berlin, Corporate Member of Freie Universität Berlin and Humboldt Universität zu Berlin, Berlin, Germany; ^6^Motognosis GmbH, Berlin, Germany; ^7^Institute of Biometry and Clinical Epidemiology, Charité—Universitätsmedizin Berlin, Corporate Member of Freie Universität Berlin and Humboldt-Universität zu Berlin, Berlin, Germany

**Keywords:** multiple sclerosis, disease progression, outcome measures, gait, balance, motor performance, Kinect, digital biomarker

## Abstract

**Background:**

This study relates to emerging concepts of appropriate trial designs to evaluate effects of intervention on the accumulation of irreversible disability in multiple sclerosis (MS). Major starting points of our study are the known limitations of current definitions of disability progression by rater-based clinical assessment and the high relevance of gait and balance dysfunctions in MS. The study aims to explore a novel definition of disease progression using repeated instrumental assessment of relevant motor functions performed by patients in their home setting.

**Methods:**

The study is a prospective single-center observational cohort study with the primary outcome acquired by participants themselves, a home-based assessment of motor functions based on an RGB-Depth (RGB-D) camera, a camera that provides both depth (D) and color (RGB) data. Participants are instructed to perform and record a set of simple motor tasks twice a day over a one-week period every 6 months. Assessments are complemented by a set of questionnaires. Annual research grade assessments are acquired at dedicated study visits and include clinical ratings as well as structural imaging (MRI and optical coherence tomography). In addition, clinical data from routine visits is provided semiannually by treating neurologists. The observation period is 24 months for the primary endpoint with an additional clinical assessment at 27 month to confirm progression defined by the Expanded Disability Status Scale (EDSS). Secondary analyses aim to explore the time course of changes in motor parameters and performance of the novel definition against different alternative definitions of progression in MS. The study was registered at Deutsches Register für Klinische Studien (DRKS00027042).

**Discussion:**

The study design presented here investigates disease progression defined by marker-less home-based assessment of motor functions against 3-month confirmed disease progression (3 m-CDP) defined by the EDSS. The technical approach was chosen due to previous experience in lab-based settings. The observation time per participant of 24, respectively, 27 months is commonly conceived as the lower limit needed to study disability progression. Defining a valid digital motor outcome for disease progression in MS may help to reduce observation times in clinical trials and add confidence to the detection of progression events in MS.

## 1. Introduction

Multiple sclerosis (MS) as a neuroinflammatory disease features a chronic course with recurrent relapses of inflammatory activity as well as chronic disability in the long-term. Accumulation of disability in multiple sclerosis may occur as relapse-associated worsening (RAW) or steady progression independent of relapse activity (PIRA) (1). While a number of MS therapies were approved to reduce the frequency of relapses, capturing disease progression in MS still poses a major challenge and an urgent scientific need. A sufficient ‘gold standard’ of clinical outcome measures in MS research and clinical practice is lacking (2) but highly desirable to assess the effectiveness of novel interventions that target chronic processes rather than relapse in this disorder (3).

A recent review reported on outcomes in phase III clinical trials of secondary progressive multiple sclerosis since 1990 (4). Among the studies reviewed, the Expanded Disability Status Scale (EDSS) (5) was by far the most frequently used outcome measure employed by 16 out of 17 trials. Increase in EDSS ratings confirmed after 3 months was most commonly used to define confirmed disability progression (CDP) events as an endpoint (6). However, there is no clear consensus on this concept and protocols diverge with respect to the time frame used to confirm EDSS progression and the cut-offs used to define increase in EDSS ratings as a function of baseline EDSS score (7, 8). The most important determinant for robust definitions of progression was the length of the confirmation period, as confirmation of the EDSS after 3 or even 6 months provided only imprecise estimates of the long-term disease course (9). Despite its extensive use in MS research, the EDSS as an ordinal scale has several limitations in terms of reliability and sensitivity (10) specifically in the early course of MS (11, 12). Another important source of variability are short-term fluctuations in performance known to occur to a relevant degree in MS (13) as well as in other chronic neurological conditions (14). Furthermore, motor performance may differ between clinical and home-based assessments (15). Thus, some of the limitations of current operational definitions of CDP are related to the fact that they rely on infrequent single-point rater-based assessments in the clinical setting. The concept of ‘No evidence of disease activity’ (NEDA) has been introduced as a potential endpoint for the evaluation of disease-modifying therapies’ (DMTs) effectiveness in relapsing remitting MS (16, 17).

Within this concept events of CDP or lack thereof within a given timeframe, respectively, represent one component. Other components are the absence of clinical relapses and absence of radiological signs of inflammatory disease activity. Recently, predictive value has been shown for total brain volume loss (BVL) on disability progression. Thus, measures of decline in brain volume have been added as a fourth component (NEDA-4) (18). Still, this concept circumvents the challenge to reliably quantify and compare the degrees of disability accumulation between subjects or treatment arms. Further, not all components can be applied in the progressive forms of MS (19).

At a time when chronic disease processes represent the target for future interventions in MS, improving the operational definitions of disability progression remains a key priority of MS research (20). Technical measures to quantify specific functions have been explored in this respect. Walking impairments are reported in up to 75% by people with MS (pwMS) (21) and thus pose a good candidate for quantitative assessment in this disorder. Consequently, the instrumental assessment of gait, mobility or other specific functions has received attention in MS research (15, 22–26). Interestingly, instrumental gait analysis has shown dysfunctional walking patterns despite clinically normal gait function even in early stage disease (27). Technical methods of remote assessment such as commercial activity trackers have a part in recent MS trials protocols (28). However, sources of variance and appropriate definitions of relevant change still need to be established for emerging digital biomarkers (29, 30).

Following this strategy, this investigation aims to devise and evaluate an instrumental definition of CDP in early RRMS by episodic patient self-assessment of motor symptoms at home. Among the various technologies available, we chose a visual-perceptive technology based on commercial RGB-D cameras. As a marker-free method this study applies a consumer-grade RGB-Depth (RGB-D) camera (Microsoft Azure Kinect®) combined with customized software (Motognosis Amsa) for motion capture at home as the primary outcome. As a marker-free method, it has high potential for clinical utility and RGB-D technology has been explored for the purpose of task-based motor assessment in various neurological conditions in research settings (31–34). As a novelty, we here turn the patient into the central operator and home-based application into the primary outcome. This enables data acquisition at higher frequency compared to conventional protocols that rely on in-patient research visits.

Primary endpoint is the accuracy of detection of progression events at 24 month compared to progression events defined as 3-month CDP in EDSS. Relation of such definitions to patients’ reports of function and impairment as well as structural change on MR imaging and optical coherence tomography (OCT) will complement the final analysis.

## 2. Methods

### 2.1. Study design

The study is a single-center prospective observational cohort study. Study data combines observations from different sources: patients’ remote self-assessment, data from treating neurologists obtained in routine clinical care—both performed semiannually—and data from annual in-person study visits at an academic clinical research center. The primary outcome consists of home-based recordings of short motor tests (Amsa, Motognosis GmbH, Berlin, Germany) performed twice a day for a period of 1 week every 6 months. Patient-reported outcomes is acquired every 6 months using validated questionnaires on specific functions, impairments and quality of life.

Data requested from treating neurologists comprise the Multiple Sclerosis Functional Composite (MSFC-3) (35), the global assessment of the change since last observation [clinical global impression of change (CGI) (36)] and information on relapse events/relapse therapy including recovery, change of therapy or comorbidity since prior visit.

Annual study visits at the research site comprise clinical and functional assessment including Motognosis Labs as well as imaging of brain (MRI) and retina (OCT) detailed below.

The observation period is 24 months for the primary endpoint with an additional clinical assessment at 27 months to confirm progression defined by EDSS.

### 2.2. Participants

The study targets a sample size of 150 people with a diagnosis of relapsing–remitting MS according to revised McDonald criteria (37) in their earlier disease course defined as <10 years since diagnosis in order to allow some heterogeneity in disability stage. Inclusion was restricted to those able to walk at least short distances with unilateral assistance at baseline—equivalent to ≤6.0 EDSS—in accordance with the requirements of the primary outcome measure. To enhance generalizability of results, study recruitment aims for at least 20% aged 55 or older and for at least 33% of EDSS ≥ 3.5 (moderately affected).

Inclusion further allows use of any intervention for MS or other morbidity as long as this is not considered to affect compliance with the study protocol. Comorbidities are not excluded as long as not considered to interfere with motor performance.

Prior disease activity or other known predictors of disease progression were implemented as additional inclusion criteria to increase expected CDP observations at 24 months while at the same time trying to maintain generalizability of our findings for the target population of early RRMS.

#### 2.2.1. Inclusion criteria

- Written informed consent to participate in this study.
- Participant’s age is ≥18 years.
- Participant resides within reasonable range from study center to allow supervision of technical set-up at home and provision of technical back-up.
- Diagnosis of relapsing–remitting multiple sclerosis according to 2017 diagnostic criteria (37).
- AND
  Disease duration of <10 years since diagnosis.
- AND
- EDSS ≤ 6.0 (ability to perform short walking tests with only unilateral assistance).
- AND
  fulfillment of one or more of the following criteria:
- history of recent disease activity: ≥ 1 relapse or ≥ 1 new T2 lesion or ≥ 1 Gd + enhancing lesion on MRI over the past 2 years.
- OR
  Findings on routine brain MRI from within 6 months prior to screening: total T2 lesion load of ≥10.
- OR
  Findings on routine brain MRI from within 6 months prior to screening: any Gd + enhancing lesion.
- OR
  Findings on routine MRI from within 6 months prior to screening: ≥ 1 spinal or brainstem lesion.
- OR
  Finding on optical coherence tomography performed at screening: Peripapillary retinal nerve fiber layer (pRNFL) < 92 μm in a non-optic neuritis eye.

#### 2.2.2. Exclusion criteria

- Relapse within 3 months prior to baseline visit.
- Other disease or condition with suspected effect on motor performance.
- Any condition foreseen to prevent compliance with protocol.
- The patient is pregnant at screening.
- Any contra-indications for MRI investigation at screening.

### 2.3. Data acquisition

An overview of the visit schedule is provided in Table 1.

**Table 1:** Overview study assessments and visit schedule.

Assessment	Rater	Screening	Baseline visit (V) 1	Visit 2	Visit 3	Visit 4	Visit 5	Visit 6
			Month 1	Month 6	Month 12	Month 18	Month 24	Month 27
Written informed consent	Investigator	□						
Assessment of in- and exclusion criteria/confirmation of in and exclusion criteria	Investigator	□	□		□		□	□
Assessment/Follow up of patients’ characteristics (diagnosis ascertainment, comorbidities, age, height, weight, relapses in past 24 months/since last visit, current symptoms, treatment: current treatment at baseline, change of treatment at follow-up)	Investigator		□		□		□	□
EDSS	Investigator	□	□		□		□	□
MSFC-4	Study assistant		□		□		□	□
6-min walk test	Study assistant		□		□		□	□
PASS-MS assessment of motor functions			□		□		□	□
PRO PGIC	Patient		□	●	□	●	□	□
PRO MSWS-12	Patient		□	●	□	●	□	□
PRO FSMC	Patient		□	●	□	●	□	□
PRO NRS	Patient		□	●	□	●	□	□
PRO HAQUAMS	Patient		□	●	□	●	□	□
PRO PHQ-9	Patient		□	●	□	●	□	□
PRO GLTEQ	Patient		□	●	□	●	□	□
PRO EQ-5D-5L	Patient		□	●	□	●	□	□
PRO BPI	Patient		□	●	□	●	□	□
Instruction (Reinstruction if needed) of use for Amsa	Study assistant		□	(●)	(□)	(●)	(□)	
Amsa assessment recorded twice a day over 7 days	Patient		●	●	●	●	●	
Reporting of AE and safety issues related to Amsa self-assessment	Patient		●	●	●	●	●	
PRO Pain-NAS, EQ-VAS, state fatigue once a day on each day of functional assessment	Patient		●	●	●	●	●	
Usability rating	Patient		●	●	●	●	●	
Brain MRI			□				□	
OCT			□				□	
Clinical global impression of change	Treating neurologist			◊	◊	◊	◊	◊
Most recent relapse incl treatment and course of remission	Treating neurologist		◊	◊	◊	◊	◊	◊
Current medication/Change in medication from baseline/comorbidities	Treating neurologist		◊	◊	◊	◊	◊	◊
MSFC-3	Treating neurologist		◊	◊	◊	◊	◊	◊

The components of each visit are denoted as (□) annual research grade assessment at study center, (●) remote self-assessment of motor functions at home (Amsa) and (◊) clinical data collected from routine visits.

#### 2.3.1. Primary outcome: self-assessment of motor functions at home (Motognosis Amsa)

Measurements are recorded with a markerless motion analysis system consisting of the measurement software (Amsa V 1.2.0, Motognosis GmbH, Berlin, Germany) running on an All-in-One PC (Optiplex 5,480, Dell GmbH, Frankfurt am Main, Germany) and a single RGB-Depth camera (Azure Kinect, Microsoft Corporation, Redmond, WA, United States).

The device is delivered to the patient’s home and set-up appropriately by qualified staff along with oral instruction of the testing protocol which includes assessment of six specified short motor tasks within the recording space of the camera. All data are stored on the system hardware only.

Participants are instructed to use Motognosis Amsa twice a day—preferably morning and later afternoon/evening—for a period of 7 days for each visit. Participants start by preparing the measuring area (e.g., removal of clutter). When the measurement area is cleared, they can start the software. The software can be controlled with gestures, i.e., lifting of the left or right arm. Assessments start with a positioning phase, where participants will see themselves on the computer screen and will be guided to the correct starting location with visual and auditory cues. Subsequently assessment-specific video instructions are provided. After execution of an assessment a result page is shown. If a measurement error occurred, a notification will be shown with the request to rerecord. Otherwise, the participant can proceed to the next assessment. If a specific task was not recorded, the participant is supposed to enter the reason for the omission in a free text field.

Assessments and their motor outcomes for this study include:

1. Stance with open and closed eyes and closed feet (20s eyes open, 20s eyes closed): angular sway speed 3D (°/s) separately for phases of stance with open and closed eyes.
2. Stepping in place (40 s): knee amplitude (m) and arrhythmicity (%).
3. Short walk in comfortable speed (Movement toward the system, stopped automatically in a certain distance): Short walk in comfortable speed: comfortable walk speed (m/s) and step width (cm).
4. Short walk in maximum speed (Movement toward the system, stopped automatically in a certain distance): maximum walking speed (m/s).
5. Line walk (Movement toward the system, stopped automatically in a certain distance): mean trunk roll deflection (°).
6. Standing up and sitting down from a chair: up time (s) and down time (s).

Performing the whole set of assessments, including in-between system operation, positioning, instructions and conduction takes at maximum 10 min. If particular assessments are deemed too risky for an individual participant by the investigator, the participant may be instructed to record only a subset of the assessments.

On each day of home-based assessment of motor function, participants are asked to answer three simple questions as potential determinants for day-to-day fluctuations: (1) about the severity of pain on a 0–10 numerical analog scale pain (painNAS), (2) about the current health status on a 0–100 visual analog scale (EQ-VAS) (38) and (3) about the current state of fatigue using a 0–10 numerical analog scale devised for that purpose. To cover aspects of patient safety, participants are reminded to report on any incidents occurring during Amsa assessment within the user interface. At the end of each week of home-based assessment, the participant is asked to fill out a questionnaire on usability of the measurement device, the System Usability Scale (SUS) Plus (39–41), translated and modified for the purpose of this project. The SUS was developed to evaluate a wide variety of products and services with a 10-item scale using five response options from “strongly agree” to “strongly disagree” to explore aspects of usability. Furthermore, participants can make suggestions how usability might be improved.

#### 2.3.2. Patient-reported outcomes

PROs listed below were applied in validated translations—except for PGIC, for which own translation was used—and completed directly in eCRF via individualized links.

##### 2.3.2.1. The patient global impression of change

The Patients’ Global Impression of Change (PGIC) scale was first developed in context of patients’ perception of changes after intervention (i.e., “feeling better” or “feeling worse”). It is a 7-point verbal scale, with the options “very much improved,” “much improved,” “minimally improved,” “no change,” “minimally worsened,” “much worsened,” and “very much worsened.” The PGIC is commonly used in clinical trials for treatments of pain, but it has also been applied as a generic measure applicable to a wide variety of conditions and treatments. Worsening of any grade is considered clinically meaningful (42).

##### 2.3.2.2. The multiple sclerosis walking scale-12

The Multiple Sclerosis Walking Scale-12 (MSWS-12) is a patient-rated measure assessing the extent to which a person’s ability to walk is affected by MS., i.e., it is conceived to capture the impairment level. It has been developed from patients’ experience and has undergone psychometric validation and translations (43). The 12 items are rated on a five-point scale (1, “not limited” to 5, “extremely”). Total scores are calculated as sum score (range 12–60) and transformed to a scale of 0–100 to aid interpretation. Higher scores reflect greater impact of MS on walking ability. An increase of >8-point in 0–100 MSWS-12 score is considered clinically meaningful (44).

##### 2.3.2.3. Fatigue scale for motor and cognitive functions

The fatigue scale for motor and cognitive functions (FSMC) is a patient questionnaire to assess MS-related cognitive and motor fatigue (45, 46). A Likert-type 5-point item rating (ranging from 1 “does not apply at all” to 5 “applies completely”) produces a sum score between 20 (no fatigue at all) and 100 (most severe fatigue). Two subscales (cognitive and physical fatigue) can be derived from the FSMC. Items included in the cognitive subscale are 1-4-7-8-11-13-15-17-18-20 and items included in the physical subscale are 2-3-5-6-9-10-12-14-16-19. An increase in FSMC category (sum score: <43: no fatigue; 43–52: mild fatigue; 53–62 moderate fatigue; >63 severe fatigue) is considered clinically meaningful (45).

##### 2.3.2.4. Spasticity using numeric rating scale

The clinical rating of spasticity will be performed using a patient-rated measure of the perceived severity of spasticity, employing a numeric rating scale (47, 48) for several aspects of spasticity, each rated on a scale of 0 to 10. Total rating is the mean of item-level answers, where 0 is no spasticity and 10 is the worst possible spasticity. Appropriate patient training has to be ensured to obtain reliable results. Any increase in 0–10 Numeric rating scale (NRS) for spasticity is considered clinically meaningful (49).

##### 2.3.2.5. Hamburg quality of life questionnaire for multiple sclerosis

The Hamburg Quality of Life Questionnaire for Multiple Sclerosis (HAQUAMS) is a health-related quality of life measure designed for pwMS (50). The HAQUAMS consists of 38 questions, 28 of which address major dimensions of health-related quality of life in MS: Fatigue/thinking (four items), mobility lower limb (five items), mobility upper limb (five items), social function (six items) and mood (eight items). Subscales and total score range from 1 to 5. Higher scores indicate a lower quality of life. Cognitive impairment in MS does not impact psychometric properties. A HAQUAMS total score increase of at least 0.22 is considered clinically meaningful worsening (51).

##### 2.3.2.6. The 9-item patient health questionnaire-9

The 9-item Patient Health Questionnaire-9 (PHQ-9) was devised to screen for depressive disorders in primary care and the setting. It is based on the DSM-IV diagnostic criteria for a major depression episode. The PHQ-9 can be used both as a screening instrument for a depressive episode and can be used to provide information about the severity of a depressive episode. Each question in the scale has four response choices: “not at all,” “several days,” “more than half the days,” and “nearly every day” and classifications of no/possibly relevant depressive disorder are made according to manual (52, 53).

##### 2.3.2.7. Godin leisure-time exercise questionnaire

The Godin leisure-time exercise questionnaire (GLTEQ) is applied here to assess physical activity levels in MS. It contains three core items regarding the frequency of strenuous, moderate, and mild physical activity for bouts of 15 or more minutes during a 7-day period (54). The scores are multiplied by weights and summed into an overall score (i.e., leisure-time physical activity [LTPA] score) that ranges between 0 and 119 metabolic equivalents of task/min of physical activity per week (55).

##### 2.3.2.8. EQ-5D/EQ—visual analog scale

EQ-5D is a generic and widely used measure of health status developed by the EuroQol Group.

Participants are asked to classify and rate their own health according to five dimensions. These dimensions comprise mobility, self-care, usual activities, pain/discomfort and anxiety. Each dimension is divided into five levels, i.e., 1 “no problems,” 2 “slight problems,” 3 “moderate problems,” 4 “severe problems,” and 5 “extreme problems.” (56). While answers on EQ-5D consider variable timeframes, the single-item EQ-VAS provides a global assessment of perceived health at the time of assessment. It consists of a vertical visual analog scale that takes values between 0 (worst imaginable health) and 100 (best imaginable health) (38).

##### 2.3.2.9. Brief pain inventory

Given the high prevalence and clinical relevance of pain, the Brief Pain Inventory (BPI) was developed as a brief instrument with low respondent burden that can be easily administered by large numbers of patients (57). The BPI measures both the intensity of pain (sensory dimension) and impact of pain with patients’ lives (reactive dimension). Pain relief, pain quality, and patient perception of the cause of pain are also addressed (58).

#### 2.3.3. Data acquisition from routine care

Information from routine care is retrieved repeatedly throughout the observation period from treating neurologists. The baseline data set comprises information on diagnosis and comorbidities, most recent relapse including its treatment and clinical outcome and full list of current medication as well as EDSS and MSFC-3, if performed routinely. Follow-up data are requested from any clinical visit throughout the observation period, at least semiannually, and comprise clinician’s global ratings of change, information on relapses since last visit in medication or comorbidities, as well as EDSS and MSFC-3.

#### 2.3.4. Data acquisition at annual study visits

##### 2.3.4.1. Study assessment

Time since diagnosis of multiple sclerosis, prior manifestations of disease, current symptoms, prior and current disease modifying therapy, supportive therapy and relapses in past 24 months as well as comorbidities and changes thereof are acquired by the clinical investigator.

Current therapy will be documented at baseline and changes thereof will be reported at each visit. This also extends to rehabilitative interventions.

##### 2.3.4.2. Expanded disability status scale

The expanded disability status scale (EDSS) is used to quantify disability in MS. The scale was first developed by Kurtzke in 1955 and then expanded in 1983. It is usually referred to as a measure which is scaled on 10 steps from 0 (no disability) to 10 (death from MS). Scoring is based on an examination by a neurologist. EDSS steps 1.0 to 3.5 refer to people with MS who are able to walk without any limitation, while EDSS steps 4.0 and higher are defined by decrease in walking capacity. Assessment of EDSS will follow instructions of Neurostatus for functional systems scores and EDSS step (59) by certified raters. In our study, the 3 m-CDP is defined as a 1.0 step increase from baseline EDSS when baseline EDSS was 0.0 to 5.0 and 0.5 step increase from baseline when baseline EDSS was 5.5 to 6.5, rated at 24 months with change confirmed at month 27.

##### 2.3.4.3. Multiple sclerosis functional composite (MSFC-3 and MSFC-4)

The MSFC was developed by a special Task Force on Clinical Outcomes Assessment as a clinically applicable standardized, quantitative assessment instrument for use in MS trials (35). The MSFC-3 measures three clinical dimensions: leg function/ambulation using the Timed 25-foot Walk test; arm/hand function using the 9 Hole Peg Test (9HPT); and cognitive function using the Paced Auditory Serial Addition Test (PASAT-3 version). Because the PASAT is not popular among patients and given the relevance of visual dysfunction in MS, an expert group (60), convened by the National MS Society, recommended two adaptations to the MSFC: (1) inclusion of the Sloan Low Contrast Letter Acuity test (60) (MSFC-4) and (2) use of the oral version of the Symbol Digit Modalities Test (SDMT) instead of the PASAT-3. In our study, the MSFC-4, will be administered on occasion of the annual visit at study site by a trained rater. In addition, the MSFC-3 will be collected from routine clinical visits semiannually. Both versions will use SDMT for the cognitive component.

The MSFC will be performed and analyzed in accordance with the respective testing manual issued by.

the National Multiple Sclerosis Society. Cut-offs for clinically meaningful change in MSFC and component Z-scores have been defined as >20% worsening (61).^1^

##### 2.3.4.4. The 6-min walk

The 6-min walk (6 MW) is applied here to measure of walking endurance/ walking capacity in pwMS (62). The test is characterized by good practicability, reproducibility and reliability in MS. Furthermore, ecological validity is supported by strong correlation to patient report of ambulation and physical fatigue (63). A ≥ 20 m decrease in distance covered in the 6-min walking test at comfortable speed is usually considered clinically meaningful (64).

##### 2.3.4.5. Supervised operator-based assessment of motor function (Motognosis Labs PASS-MS)

Our group developed a clinically applicable assessment protocol (PASS-MS) for usage with the Motognosis Labs system (Motognosis GmbH, Berlin, Germany). Motognosis Labs functions similar to Amsa in terms of technology. It differs *in camera* version used (Microsoft Kinect v2 for Motognosis Labs vs. Azure Kinect for Motognosis Amsa) and test set-up, as Motognosis Labs uses a camera plugged in to a laptop and participants are guided through assessments by a trained operator according to standard instructions.

PASS-MS consists of 10 short motor tasks performed in front of the RGB-D camera and parameters for the description of performance are generated by custom scripts. In operator-based application, this system proved acceptable to patients and was easily applied. Previous validation showed accuracy of derived parameters against gold standard multi-camera motion capture (33) and sufficient reliability and validity to measure balance and gait function in MS (65, 66).

Assessment of PASS-MS includes:

1. Stance with open and closed eyes and closed feet (20s eyes open, 20s eyes closed).
2. Dual Task Stance with open and closed eyes and closed feet.
3. Stepping on place (40s).
4. Short walk in comfortable speed.
5. Short walk in maximum speed.
6. Line walk.
7. Standing up and sitting down from a chair.
8. Pronator drift test.
9. Finger-to-nose-test.
10. Finger tapping.

##### 2.3.4.6. Magnetic resonance imaging

In this study a standardized MR protocol was performed consisting of: a 3D-T1-weighted sequence (MPRAGE), a 3D T2-SPACE, a 3D fluid attenuated inversion recovery (FLAIR), a Diffusion Weighted Imaging sequence (DWI) and a resting state functional MRI (rsfMRI). To define progression, we use operational cut-offs for brain volume loss known to increase with age. Mean BVL per year amounts to by 0.15, 0.30, 0.46, and 0.61% of baseline brain volume at ages 45, 55, 65, and 75 years, respectively (67). The corresponding age-dependent 95th percentiles of BVL per year were 0.52%, 0.77%, 1.05%, and 1.45%. Pathological BVL can be assumed if an individual BVL per year exceeds these thresholds for a given age (67).

##### 2.3.4.7. Optical coherence tomography

Optical coherence tomography (OCT) is a suitable high-resolution imaging method for the assessment of retinal integrity with good reproducibility. Peripapillary retinal nerve fiber layer thickness and macular volume are the most reported indicators to measure retinal atrophy on OCT. It has been shown that pwMS with a pRNFL thickness of less than or equal to 87 μm (88 μm) measured with Spectralis (Cirrus) OCT devices had double the risk of disability worsening in the follow-up (68).

which led us to consider this measure as a component of the inclusion criteria. With respect to progression of structural abnormalities of the retina, an absolute thinning of pRNFL of more than 1.25 μm at 24 months against baseline is considered clinically meaningful. This threshold has been established previously as the upper limit to define stability in multiple sclerosis (69).

#### 2.3.5. Visit schedule

See Table 1.

### 2.4. Pre-processing of data

All study data will undergo plausibility checks including description of missings prior to further analysis. Definitions of progression events by EDSS or alternative definitions for secondary outcomes are applied as provided in section 2.3.

Data from technical recordings (Amsa, Motognosis Labs, MRI, and OCT) are continuously monitored by trained users to check usability and plausibility according to standard operating procedures for quality control (QC). Specifically, for Amsa and PASS-MS, presentations of all assessments are systematically inspected and evaluated for quality concerns following the QC pipeline developed by Röhling et al. (70).

For RGB-D camera based motion capture, both Amsa and PASS-MS, a list of task-specific kinematic parameters is generated according to custom scripts by the provider. Kinematic parameters include for example gait speed for the short walking tasks. Primary analysis will consider only one parameter per test condition, i.e., seven parameters.

### 2.5. Data management

**Figure 1:**
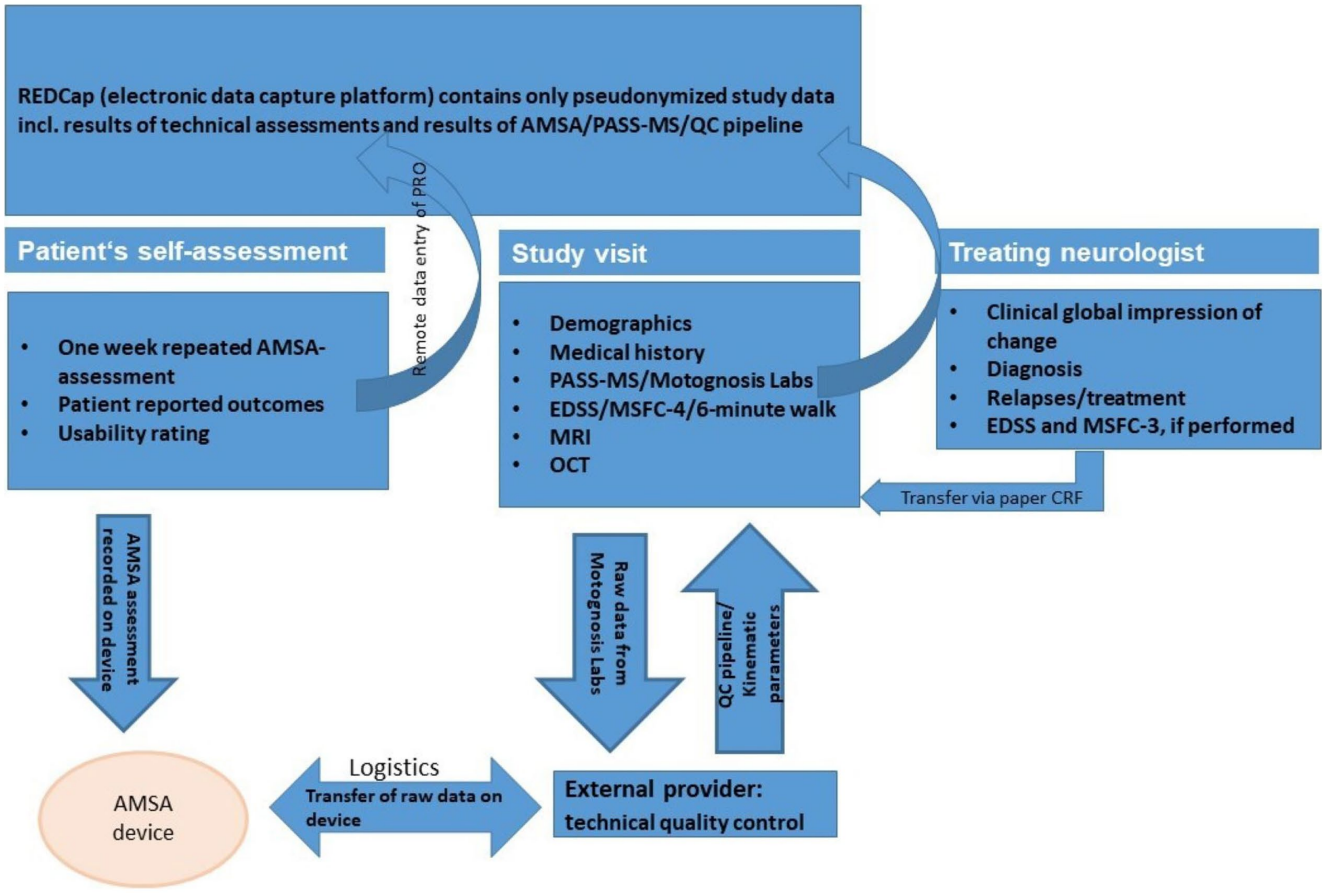
Data flow.

Data flow is shown in Figure 1. Study data are stored in an study-specific Electronic Data Capture platform (REDCap) (71). The platform also enables remote data entry of PRO by patients themselves via personalized links. The study team provides the links to each participant at the appropriate timepoints throughout the observation period.

For the technical assessments performed in this study (Amsa, PASS-MS/Motognosis Labs, MRI, OCT), test results (e.g., gait speed from PASS-MS, number of T2 lesions on MRI, pRNFL thickness in OCT) are only transferred into REDCap system after pre-processing as described in section 2.4 along with results of QC.

All pseudonymized raw data are digitally stored in separate archives on local platforms.

With regard to data imports from home-based repeated Amsa assessments, raw data and metadata are saved on and retrieved with the device after each 1-week assessment period by the provider. The provider, after checking for technical issues and completeness, transfers results of daily self-ratings and pseudonymized metadata and raw data from Amsa recordings to the study site. Data are provided for QC application as well as kinematic parameters for analysis and archiving.

Data from routine care are collected from treating neurologists using structured paper templates.

### 2.6. Status of the study

Approval of the institutional review board (Charité—Universitätsmedizin Berlin) was obtained on 21 October 2021 (EA1/293/21). The study is active with first patient first visit in June 2022.

### 2.7. Statistical analysis plan

Based on the study question, the primary hypothesis is to assess the accuracy of detecting disease progression by the repeated short motor assessments at 24 months, compared to progression defined by EDSS at 24 months and confirmed at 27 months.

The sample size is justified via the expected precision of the estimate of the area under the receiver operating characteristic curve (AUC) provided in terms of the 95% confidence interval. We hypothesize that the parameters derived from repeated short motor assessment at home can reliably identify disease progression compared to detection of progression according to EDSS.

If the AUC is 0.9, and the sample size is 150, from which 10% are diagnosed with a progression according to EDSS at 24 months, then the width of the 95% confidence interval will be 0.103. In case of a lower AUC, e.g., 0.8, the 95% confidence interval would be of width 0.138. These calculations were performed using nQuery version 8.7.0.0, procedure AOC6-1. Given these calculations, the expected precision of the estimated quantity seems sufficient to evaluate the exploratory research hypothesis of this study.

Given the baseline definition, the primary endpoint is analyzed as AUC of detection of disease progression according to the repeated short motor assessment at home compared to detection of disease progression according to the EDSS score at 24 months, confirmed at 27 months. The AUC is calculated alongside with the 95% confidence interval. Event of progression at 24 months detected by parameters from repeated short motor assessment at home is defined as observed reliable change in pre-defined direction of worsening in one or more of the parameters derived from this assessment. Threshold for reliable change will be defined based on analysis of baseline data. Secondary endpoint analyses are planned for MSWS-12, HAQUAMS, PGIC, CGI, MSFC, and 6 MW. Respective receiver operating characteristic curves and their AUC will be calculated based on published evidence on minimally important difference for MSFC, 6 MW, MSWS-12, and HAQUAMS while any transition in the direction of worsening will be considered an important change on PGIC and CGI.

Subgroup analysis are planned for stratification by age > 55 years vs. younger and EDSS < 3.5 vs. higher. Further exploratory subgroup analysis may be conducted for all endpoints, if there exist relevant subgroups, using standard statistical methods such as parametric or non-parametric location tests and regression methods.

## 3. Discussion

This study evaluates the concept of remote multipoint assessment of motor performance to improve definitions of disease progression in MS over current definitions of 3 m-CDP. Being among the first and larger studies to evaluate quantitative motor assessments longitudinally (28, 72–76), it is also the first study to apply RGB-D technology for the remote assessment of motor functions in MS (77). The prospective design and multimodal examination protocol ensures that changes defined by remote assessment can be compared with state-of-the-art clinical and imaging endpoints in MS.

Primary analysis will use 3 m-CDP defined by EDSS as the main comparator, which is most commonly used as an endpoint of disability accumulation in MS clinical trials to date. Still, more general concerns have been raised with respect to disability confirmation by EDSS (78). For example, 3 m-CDP may overestimate the accumulation of disability in the longer term and, thus, longer confirmation periods should be preferred (79) which would extend observation times. We here adhere to an observation period of 24 months as the minimum among previous studies that used CDP as their main endpoint. Within this timeframe, reasonable proportions of progression events can be expected according to previous reports. Nonetheless, proportions were not much higher than 10% in earlier disease stages (80–82). Targeting this group for our study has the risk that progression observed is too subtle to yield numbers that could answer the primary research question. Therefore, the inclusion criteria were set to increase the likelihood of disability progression while at the same time maintaining generalizability for early MS.

The technical approach applied in this study is novel in two aspects: first, assessments of motor function are recorded by patients themselves in their home setting, and second, this approach enables frequent (multi-point) assessment. Both aspects hold potential to overcome the limitations of conventional rater-based assessments in single and infrequent clinical visits. Previous evidence suggests that different features of MS may vary considerably within the same subject, including self-ratings of health status (83). For fatigue, over one-third of the variability can be attributed to moment-dependent fluctuations, 8.2% to day-to-day fluctuations, while 56.6% can be attributed to individual differences (84) and many pwMS report increased fatigue in the afternoon and evening (85). Day-to-day variability has also been shown for maximum walking distance that would equal changes of up to 1.5 EDSS points (86). We therefore expect to observe day-to-day variability of motor performance throughout each week of remote assessment and consider this in definitions of change at follow-up. Further, our study protocol combines repeated self-recording of motor functions with a daily self-report of health status, pain and state fatigue. This will allow us to study possible correlates.

For a quantitative assessment of motor functions in MS, this study follows the technical approach of a task-based assessment. Clinical relevance of instrumentally assessed gait quality has been shown in early-stage multiple sclerosis (87). However, their ecological validity may be limited, depending on task and setting (88).

Shema-Shiratzky et al. found that dual-task walking in the lab better represents walking ability in everyday life, whereas usual walking in the lab is more likely to represent best performance in everyday life (89).

We therefore included additional performance-based and patient-reported outcomes of walking function for contextualization. Another novelty of our study is the integration of data acquired semiannually in the course of routine clinical care by treating neurologists. Analysis will use these, specifically their global ratings of change, as one of the alternative secondary definitions of change. This intends to explore the validity and generalizability of our findings for the setting, in which approved treatments or digital motor applications will ultimately be applied.

We aimed to address potential drawbacks of remote task-based assessment in study design and analysis plan. First, variability in task performance can be expected to be higher in unsupervised settings. We aim to mitigate this point by control of device set-up by a trained operator, standardized instructions and by technical design. Second, we apply a standardized quality control pipeline on recordings to identify relevant protocol deviations. Roehling et al. showed the feasibility but also the necessity for a *post hoc* quality control using this method of instrumented motion analysis (70). In this study, we extend this QC pipeline exploring its practicality to Amsa measurements. Furthermore, participants’ adherence is surely a concern in this long-term observation. A recent 8-week RCT study investigated participants’ short term adherence in using digital tools in multiple sclerosis. The average overall adherence for all three measurement tools (1. MS patient-reported outcome tool accessible via a smartphone app, 2. Floodlight open, a app-based assessment of hand and gait function, and 3. Fitbit, a smart watch for passive monitoring of sleep duration and quality) was 81% in the intervention group (90). Midaglia et al. analyzed the practicability of remote active testing and passive monitoring using digital tools in pwMS and showed 70% (16.68/24 weeks) adherence to active testing and 79% (18.89/24 weeks) to passive monitoring (74). Rates of attrition in application of remote assessment have not yet been reported over longer time-frames. In order to sustain adherence in our 24 months-study, participants will receive a reminder link about 4 weeks before the upcoming measurement and the Amsa device will be delivered by the study team at the appropriate time.

Our aim is to evaluate subtle changes in motor signs to identify chronic progression in MS independent of relapse by remote task-based assessment. This new approach will be related to the EDSS as well as imaging surrogates (OCT, MRI). Reliable remote assessment of disability would seamlessly fit in the landscape of digital health solutions that are highly important in situations where specialized care is scarce or episodically unavailable, such as in recent pandemic conditions. If utility can be shown for this home-based setting, such assessment may serve as a valuable source of information in patient care. An appropriate re-definition of progression events may substantially reduce total observation times and rater involvement in clinical trials that aim to establish clinical stability or clinical progression in MS as their outcome of interest.

## Ethics statement

The study was approved by the Institutional Review Board (Ethikkommission Charité Universitätsmedizin Berlin, EA1/293/21). Participants are enrolled only after written informed consent. The study was registered at Deutsches Register für Klinische Studien (DRKS00027042). Dissemination includes this submission of the study protocol for peer-reviewed publication and discussion of interim and final results at conferences as well as prospective publication in peer-reviewed journals.

## Author contributions

E-MD: Conceptualization, Data curation, Investigation, Methodology, Project administration, Supervision, Writing – original draft, Writing – review & editing. HR: Conceptualization, Data curation, Software, Writing – review & editing. DZ: Conceptualization, Formal Analysis, Writing – review & editing. LH: Conceptualization, Formal Analysis, Writing – review & editing. FP: Supervision, Writing – review & editing. TS-H: Conceptualization, Data curation, Funding acquisition, Investigation, Methodology, Project administration, Supervision, Writing – original draft, Writing – review & editing.

## Footnotes

^1^ http://main.nationalmssociety.org/docs/HOM/MSFC_Manual_and_Forms.pdf

## Glossary

AUC: Area under the receiver operating characteristic curve
BVL: Brain volume loss
BPI: Brief pain inventory
CDP: Confirmed disability progression
CGI: Clinical global impression of change
DMTs: Disease modifying therapies
EDSS: Expanded disability status scale
FSMC: Fatigue scale for motor and cognitive functions
GLTEQ: Godin leisure-time exercise questionnaire
HAQUAMS: Hamburg quality of life questionnaire for multiple sclerosis
MRI: Magnetic resonance imaging
MS: Multiple sclerosis
MSFC: Multiple sclerosis functional composite
MSWS-12: The multiple sclerosis walking scale-12
NAS: Numerical analog scale
NEDA: No evidence of disease activity
NRS: Numeric rating scale
OCT: Optical coherence tomography
PASAT: Paced auditory serial addition test
PGIC: Patients’ global impression of change
PHQ-9: Patient health questionnaire-9
pRNFL: Peripapillary retinal nerve fiber layer
PRO: Patient reported outcome
pwMS: People with multiple sclerosis
QC: Quality control
RCTs: Randomized controlled trials
REDCap: Research electronic data capture
RGB-D: RGB-D camera that provides both depth (D) and color (RGB) data
RRMS: Relapsing–remitting multiple sclerosis
SDMT: Symbol digit modalities test
SUS-Plus: System usability scale plus
V: Visit
VAS: Visual analog scale
VPC: Visual perceptive computing
3 m-CDP: 3-month confirmed disease progression
6 MW: 6-min walk
